# Supplementary material for: Exploratory adaptation in large random networks
Source: Nat Commun. 2017 Apr 21;8:14826. doi: 10.1038/ncomms14826 (PMC5413947; doi:10.1038/ncomms14826)
Supplement: Supplementary Information — Supplementary figures, supplementary notes and supplementary references. [file ncomms14826-s1.pdf]

Supplementary Note 1

Empirical Spectrum of Interaction Matrices W

The initial interaction matrix $J_0 \triangleq J(t = o)$ is defined as a random Gaussian matrix with mean 0 and variance $\frac{{g_0}^2}{\langle K \rangle}$, where $\langle K \rangle$ is the mean in and out degree, and g_0 (the network gain) determines the spectral radius of the combined interaction matrix W at $t = 0$ (see Methods section in main text). Empirically we find that for matrices of relevant size the spectral radius of W is not greatly affected by topology and it remains $\sim g_0$ following the above normalization $Var(J_{0ij}) = \frac{{g_0}^2}{\langle K \rangle}$, however the distribution is highly non-uniform (Sup. Fig. 1)

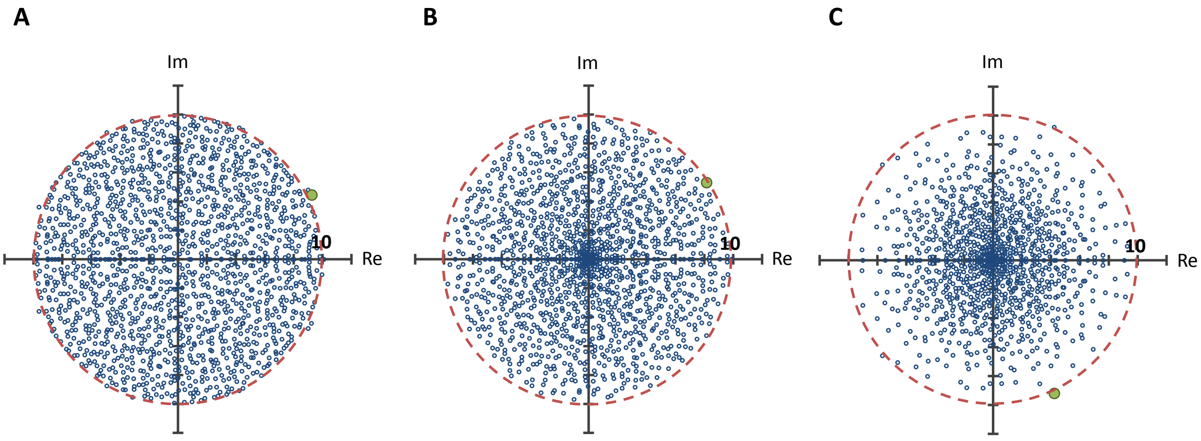

Supplementary Figure 1. Eigenvalues of finite size matrices with N=1500. The eigenvalues of full Gaussian (A), sparse Gaussian (B) and scale-free/binomial (C) matrices are plotted. The eigenvalues of all three matrices are almost entirely contained within a disc of radius g_0 (broken red) and all three have a largest norm of eigenvalue $\sim g_0$ (green dot). However, the distribution of eigenvalues in the disc differs considerably between the three matrices. The number of non-zero elements in the sparse Gaussian matrix (B) is distributed with Binomial distribution in both columns and rows. The scale-free/binomial matrix (C) has a Binomial distribution for the number of non-zero elements in the rows and a scale-free distribution in the columns. All matrices have the form $W = T \circ J$, with $J_{ij} \sim \mathcal{N}(0, \frac{{g_0}^2}{\langle K \rangle})$ and $g_0 = 10$. Binomial distributions in (B) and (C) have parameters $p \simeq \frac{5}{N}$ and scale-free distribution in (C) has parameters $a = 1$, $\gamma = 2.2$.

Distributions of Phenotype y

The variable representing the macroscopic phenotype is defined as $y(\mathbf{x}) = \mathbf{b} \cdot \mathbf{x}$. The arbitrary weight vector $\mathbf{b}$ is characterized by a degree of sparseness c, i.e. the fraction of nonzero components, $\frac{1}{N} < c < 1$.

The non-zero components of $\mathbf{b}$ are thus distributed $b_i \sim \mathcal{N}(0, \frac{1}{{g_0}^2 \cdot cN} \cdot \alpha)$ (see Methods section in main text). Sup. Fig. 2(A-C) depicts distributions of the values of y for $\alpha = 100$ with various types of interaction matrices W. As can be seen, these distributions are similarly shaped for a broad range of network sizes (Sup. Fig. 2A) and gains, g_0 (Sup. Fig. 2B), and do not change for various network topologies (Sup. Fig. 2C). These results verify that y and J_0 are appropriately normalized.

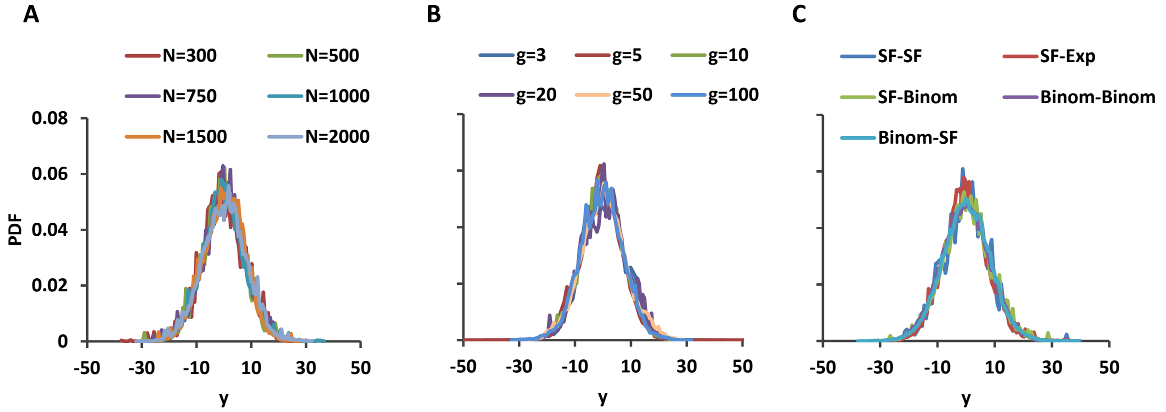

Supplementary Figure 2. Distributions of phenotype y over trajectories for various network ensembles The distribution of the values of the macroscopic phenotype $y = \mathbf{b} \cdot \mathbf{x}$ are plotted for various ensembles of networks with fixed interaction strengths. These include networks of various sizes (A), network gains (B) and topologies (C). For all ensembles the y values are similarly distributed. This indicates that y and J_0 are appropriately normalized. For all networks $\alpha = 100$. Networks in (A) and (B) have Scale-free out-degree distribution and Binomial in-degree distribution; Networks in (A) and (C) have $g = 10$ and networks in (B) and (C) have $N = 1000$. In all panels scale-free in/out distributions have parameters $a = 1$ and $\gamma = 2.4$, exponential distributions have parameter $\beta = 3.5$ and binomial distributions have parameters $p = \frac{3.5}{N}$ and N.

Convergence of Different Network Ensembles

The topological ensembles in our model includes both quenched and annealed disorder. The random topology of the network, namely the specific adjacency matrix T , is quenched and remains the same throughout the course of any single simulation run. The strengths of the network interactions, $J(t)$, on the other hand, are dynamic and change via a random walk, thus presenting an annealed disorder. Convergence fractions are computed by averaging over such simulations; one needs to determine what is the relevant ensemble to average over.

Given a choice of the model parameters, one possible ensemble $\{(T^j, J_0^j, \mathbf{x}_0^j)\}_{j=1}^m$, consists of a set of m networks, each with a different topology T^j, different initial interaction strengths $J_0^j \triangleq J^j(t=0)$ and different initial conditions $\mathbf{x}_0^j \triangleq \mathbf{x}^j(t=0)$. Another potential ensemble, $\{(T^0, J_0^j, \mathbf{x}_0^j)\}_{j=1}^m$, contains of a set of networks which all share the same adjacency matrix T^0, but differ in the initial network strengths J_0^j, and initial conditions $\mathbf{x}_0^0$; A third possibility is constructing an ensemble by varying only the initial conditions $\mathbf{x}_0^j$ and using the same initial network $W^0 = T^0 \circ J_0^0$, $\{(T^0, J_0^0, \mathbf{x}_0^j)\}_{j=1}^m$, and finally, one can simulate the dynamics consecutively keeping both the initial network W^0 and initial dynamical conditions

$\mathbf{x}_0^0$ constant, $\{(T^0, J_0^0, \mathbf{x}_0^0)\}_{j=1}^m$, with different realizations of the exploration process. Whether or not these various ensembles show qualitatively similar statistical properties or not is *a-priori* known and depends on the self-averaging properties of the system.

We tested these properties by computing the distribution of convergence times for the various ensembles. Sup Fig. 3 shows that these distributions are similarly shaped for all ensembles.

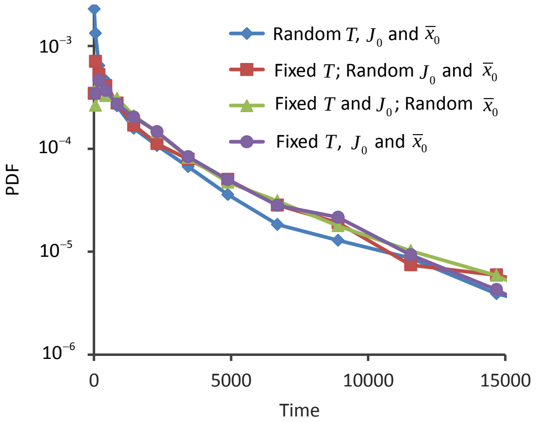

Supplementary Figure 3 Convergence Time distributions for Different Network Ensembles. (i) An ensemble in which each network has random T^j, J_0^j and $\mathbf{x}_0^j$ (blue); (ii) An ensemble in which all networks share the same topology T^0, but differ in J_0^j, and $\mathbf{x}_0^j$ (red); (iii) An ensemble in which initial network $W = T^0 \circ J_0^0$ is the the same for all networks but initial conditions $\mathbf{x}_0^j$ are unique (green) (iv) An ensemble in which both the initial network W^0 and the initial dynamical conditions $\mathbf{x}_0^0$ are the same for all networks. All Ensembles have SF out-degree distribution and Binomial in-degree distribution. The backbone T is the same matrix for ensembles (ii), (iii) and (iv) and the initial interactions strengths J_0 is the same in ensembles (iii) and (iv). For all ensembles $N = 1500$, $g_0 = 10$, $\alpha = 100$, $\mathcal{M}_0 = 2$, $c = 0.2$, $\varepsilon = 3$, $D = 10^{-3}$ and $y* = 0$. SF out-degree distribution has parameters $a = 1$, $\gamma = 2.2$, and Binomial in-degree distributions has parameters $p = \dfrac{5}{N}$ and N.

Supplementary Note 2

Robustness of Model to Saturating Function ϕ

The dynamics of the microscopic variables $\mathbf{x}$ prior to any exploration in W is given by

$$\dot{\mathbf{x}} \;=\; W\phi(\mathbf{x}) - \mathbf{x}. \tag{1}$$

The results shown in the main text were obtained using the element-wise saturating function $\phi(x_i) = \tanh(x_i)$. However, we find that these main results hold also for other types of saturating functions, specifically piece-wise linear and Sign function. In all cases convergence fractions depend on the topology of the networks, with higher fractions for those with scale-free out-degree distribution (Sup. Fig. 4A,B). The slope of the saturating function at zero has little impact on convergence fractions (Sup. Fig. 4C,D)

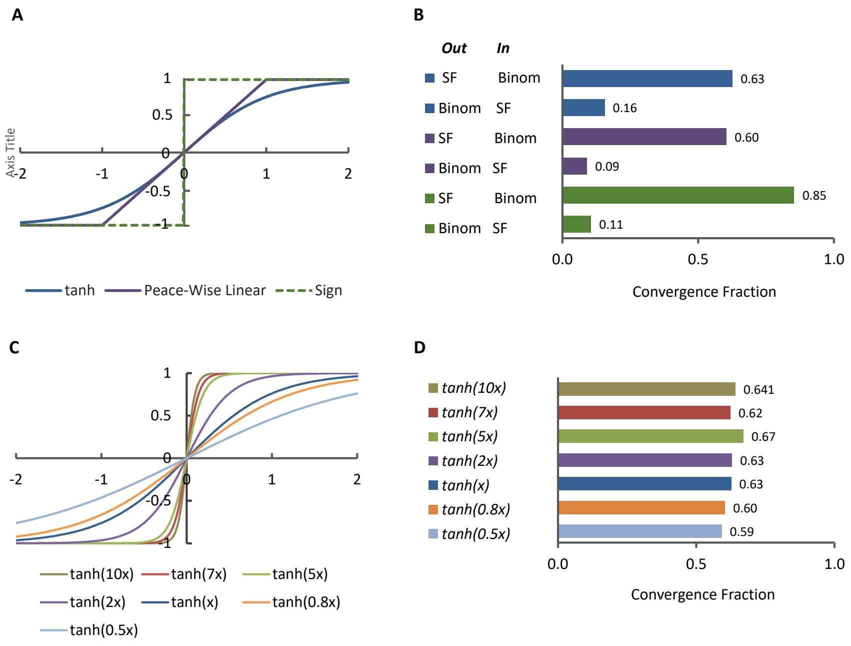

Supplementary Figure 4. Convergence fractions for dfferent saturating functions. (A) Functional from of three saturating functions examined: $\tanh(x_i)$ (blue), piece-wise linear (purple) and Sign function (broken green) (B) Convergence fractions for ensembles with the three functional forms and two types of topology SF-Binom and Binom-SF within a time window of 2000 units .(C) Functional from of saturating functions with various slops at zero. (D) Convergence fractions for ensembles with functional forms of ϕ shown in (C) with SF-Binom topology, within a time window of 2000 units. The ensemble samples in (B) and (D) consist of 500 networks each. For all networks $g_0 = 10$, $\alpha = 100$, $c = 0.2$ $\mathcal{M}_0 = 2$, $D = 10^{-3}$ and $\varepsilon = 3$, $y* = 0$. Scale-free in/out distributions have parameters $a = 1$ and $\gamma = 2.4$, $\beta \sim 3.5$ and Binomial distributions have parameters $p \simeq \dfrac{3.5}{N}$ and $N = 1000$.

Robustness of Model to Position of Saturating Function ϕ

In the model described in the main text the saturating function $\phi(x_j)$ operates directly on x_i prior to the interactions, while the interactions w_{ij} multiply $\phi(x_j)$ (See Eq. 1 above). However, it is of interest to examine a possible alternative model in which the saturating function ϕ operates on $W\mathbf{x}$ and the equation of motion is

$$\dot{\mathbf{x}} = \phi(W\mathbf{x}) - \mathbf{x}. \tag{2}$$

or equivalently

$$\dot{x_i} = \phi\left(\sum_j W x_j\right) - x_i. \tag{3}$$

Similar equations are often used to describe the dynamics of neural networks, as well as gene interactions. It is not *a-priori* whether these two formulations will result in similar convergence properties in the context of the exploratory adaption protocol described here. Remarkably, we find convergence fractions of the two models to be almost identical (Sup. Fig. 5), as long as the macroscopic phenotype y is appropriately normalized (see Sup. Fig. 2). Recall that for the model described in the main text the elements of the vector $\mathbf{b}$ which defines the phenotype y are given by $b_i \sim \mathcal{N}(0, \frac{1}{{g_0}^2 \cdot cN} \cdot \alpha)$. The normalizing factor $\frac{1}{{g_0}^2 \cdot cN}$ ensures $y \sim \mathcal{N}(0, \alpha)$ prior to convergence. The variance of b_i is normalized by $\frac{1}{{g_0}^2}$ due to the empirical distribution of x_i prior to convergence: $x_i \sim \mathcal{N}(0, \sim {g_0}^2)$. In the alternative model described by Eqs. 2,3 this is not the case. For $g_0 >> 1$, x_i mostly attains the saturated values of ϕ which are ± 1 with equal probability and $Var(x_i) \sim 1$. Thus the normalizing factor $\frac{1}{{g_0}^2}$ can be dropped and $b_i \sim \mathcal{N}(0, \frac{1}{cN} \cdot \alpha)$ results in $y \sim \mathcal{N}(0, \alpha)$ as in the former case.

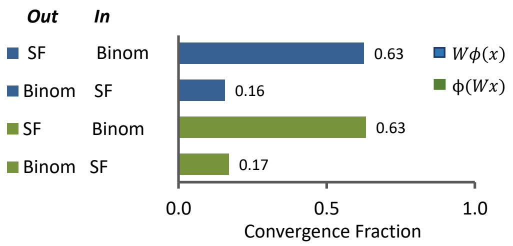

Supplementary Figure 5. Convergence Fractions with saturating functions inside and outside the summation. Convergence fractions within a time window of 2000 units for the model described in the main text (Blue) and a similar model in which the saturating function is placed outside the summation (Green). Convergence Fractions are shown for ensembles with two types of topology: SF-Binom (out-in) and Binom-SF (out-in) for both models. For all networks $g_0 = 10$, $\alpha = 100$, $c = 0.2$ $\mathcal{M}_0 = 2$, $D = 10^{-3}$ and $\varepsilon = 3$, $y* = 0$. Scale-free in/out distributions have parameters $a = 1$ and $\gamma = 2.4$, $\beta \sim 3.5$ and Binomial distributions have parameters $p \simeq \frac{3.5}{N}$ and $N = 1000$.

Robusntess of Model to Mismatch function $\mathcal{M}(y)$

For all computations shown in the main text, the mismatch function $\mathcal{M}(y)$ is defined as a symmetric sigmoid around $y*$

$$\mathcal{M}(y) = \frac{\mathcal{M}_0}{2}\left[1 + \tanh\left(\frac{|y - y*| - \varepsilon}{\mu}\right)\right], \tag{4}$$

where 2ε is the size of the low mismatch comfort zone around zero, μ controls the steepness of the sigmoid in its dynamic range, and $\mathcal{M}_0$ is its maximal value (see Sup. Fig. 6, blue line). An alternative linear mismatch function

$$\mathcal{M}(y) = \begin{cases} |y - y*| - \varepsilon & |y - y*| > \varepsilon \\ 0 & |y - y*| \leq \varepsilon \end{cases} \tag{5}$$

was examined (see Sup. Fig. 6, red line), resulting in similar convergence properties. However, using a parabolic function for the mismatch resulted in poor convergence fractions for the same parameters displayed in the main text. Thus, the existence of a broad region of zero mismatch, rather than a well-defined minimum at a point, seems essential for convergence by exploratory adaptation, but the detailed shape of the function does not seem to have a large impact on the results.

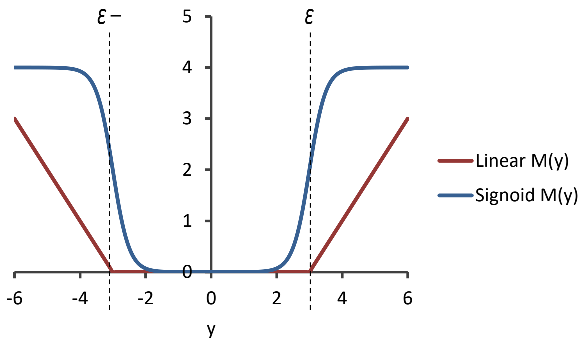

Supplementary Figure 6. Mismatch Functions Two examples of mismatch functions $\mathcal{M}(y)$ that result in similar convergence behavior. The results shown in the main text and supplementary were obtained using a sigmoidal mismatch function (blue); similar convergence results can be obtained with a linear mismatch function (red) as well (convergence results not shown). The sigmoidal function in the figure has parameters $\varepsilon = 3$, $mu = 0.5$ and $\mathcal{M}_0 = 4$.

Supplementary Note 3

Convergence to a Limit Cycle

An example of convergence to a fixed-point which satisfies the constraint is shown in the main text (Fig. 1 B-D). However, the non stringent constraint which is reflected in the "comfort zone" of the mismatch function $\mathcal{M}(y)$ allows for a time-varying solutions with small amplitude which are not fixed-points. Indeed, many simulations converge to a limit cycle solution (example shown in Sup. Fig. 7 A,B). Such a solution satisfies the constraint only if the amplitude of macroscopic phenotype y is confined in the range $(-\varepsilon, +\varepsilon)$ (Sup. Fig. 7A). The microscopic variables x_i also converge to limit cycles, but these can vary in amplitude and values (Sup. Fig. 7B). Interestingly for a broad range of network sizes and different network topologies the ratio between convergence of exploratory dynamics to fixed-points and to limit-cycles is largely preserved. For the ensembles shown in Sup Fig. 7C roughly 35% of the solutions are limit cycles and the rest are fixed-points.

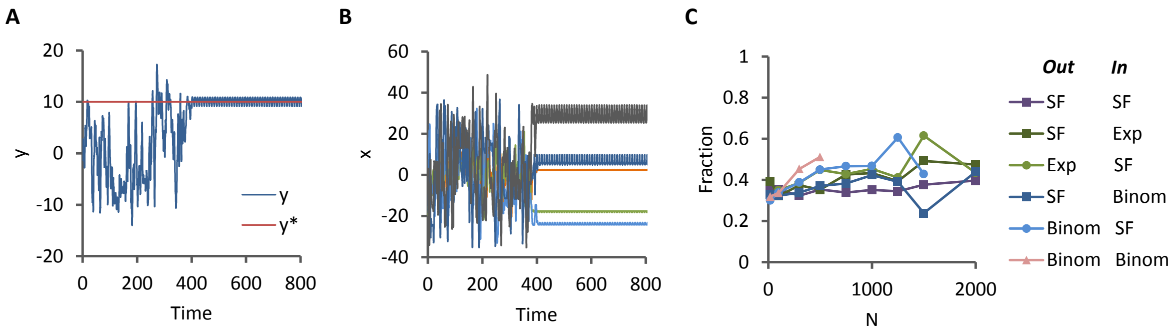

Supplementary Figure 7. Convergence to Limit Cycles. (A) The macroscopic phenotype y as a function of time in one simulation which converged to a small-amplitude limit tycle around y^*. (B) Several microscopic variable x_i as a function of time in the same simulation; x_i also converged to limit cycles but with various amplitudes and centers. (C) Fraction of networks that converged to limit cycles within a time window of 2000 time units, as a function of network size. Results are shown for different ensembles, each composed of a sample of 500 networks. Networks in each of the ensembles has a random T, J_0 and $\mathbf{x}_0$. The network in (A) has SF out-degree and Binomial in-degree distributions. For all networks in (A) (B) and (C) $g_0 = 10$, $\alpha = 100$, $c = 0.2$ $\mathcal{M}_0 = 2$, $D = 10^{-3}$ and $\varepsilon = 3$. In (A) and (B) $y* = 10$ and in (C) $y* = 0$. In all panels scale-free in/out distributions have parameters $a = 1$ and $\gamma = 2.4$, exponential distributions have parameter $\beta \sim 3.5$ and Binomial distributions have parameters $p \simeq \dfrac{3.5}{N}$ and N.

Dependence of Convergence in Scale-Free Networks on Pareto Distribution Parameters

As mentioned above, scale-free degree distributions were sampled by discretesizing the continuous Pareto distribution

$$P(k) = \frac{(\gamma - 1)a^{\gamma-1}}{k^{\gamma}}, \tag{6}$$

where the parameter a controls the minimal value of the support and γ controls the power law tail of the distribution. In contrast to directly sampling from a discrete distribution such as the Zeta distribution, such a sampling method allows additional control of the lower part of the distribution. After discretization the minimal possible degree, k_{min} is the integer which is nearest to a regardless of non-integer values assigned to a. However, the exact value of a affects the weight of the distribution at its minimal value k_{min} and the overall shape of the discrete distribution at its lower part. For example, for every $a \in [1, 1.5)$ the minimal degree in the network would be 1. However for $a = 1.1$ there would be a higher probability for nodes with degree 1 then with $a = 1.4$. This allows us to examine with detail the effect of the lower part of the scale-free distribution on the convergence properties of the model. We find that convergence of exploratory adaptation is indeed sensitive to the the weights at the lower part of the out-going distribution (Sup. Fig. 8A), and occurs with high fractions only for networks with a large enough number of nodes with out-going degree 1 ($a \in (0.4, 1.4)$). In contrast, convergence is weakly dependent on the exact power law of the distribution γ (Sup. Fig. 8B).

While a and γ have a very different effect on the distribution, they both influence the mean degree $\langle k \rangle$ of the network. Sup Fig. 8C shows the same convergence fractions plotted as a function of the mean degree. The results indicate that $\langle k \rangle$ does not directly influence convergence fractions, and highlights the sensitivity to the lower-part of the distribution which is controlled by a. Recent findings have shown that the controllability of random networks is strongly affected by the minimal degree of the nodes with a transition when the minimal degree is increased to $k_{min} \geq 2$ [1]. For our model we conclude that both the existence of hubs and the existence of a large number nodes with out-going degree 1 are indicative of convergence to a stable state.

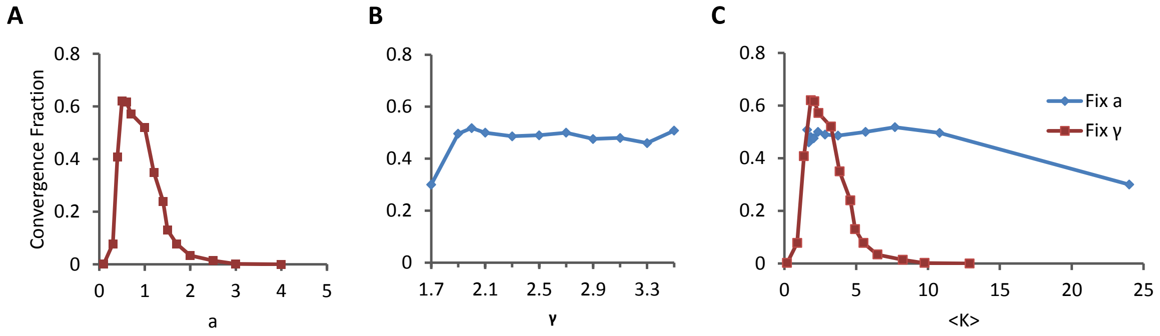

Supplementary Figure 8. Dependence of convergence fractions on parameters of out-degree Pareto Distribution. Scale-free degree distributions are sampled by discretesizing the continues Pareto distribution (Eq. 6). (A) Convergence fracion as a function of a, a parameter which controls the lower part of the distribution. (B) Convergence fraction as a function of γ, which controls the power-law tail of the distribution. (C) Convergence fraction as a function of mean degree $\langle K \rangle$; changes in this mean degree can be obtained by varying either a (red line) or γ (blue line). Each data point in (A), (B) and (C) represents the fraction of network which converged within a time window of 2000 time units from a different ensemble of 500 networks. Networks in each of the ensembles has a random T, J_0 and $\mathbf{x}_0$. All Ensembles have SF out-degree distribution and Binomial in-degree distribution. For all results $N = 1000$, $g_0 = 10$, $\alpha = 100$, $c = 0.2$ $\mathcal{M}_0 = 2$, $\varepsilon = 3$, $D = 10^{-3}$ and $y* = 10$. Scale-free out-degree distributions have parameters $a = 1$, $\gamma = 2.4$, and Binomial in-degree distributions have $p = \frac{3.5}{N}$.

Dependence of Convergence on Sparseness of Macroscopic Phenotype

As mentioned above the macroscopic state, $y(\mathbf{x}) = \mathbf{b} \cdot \mathbf{x}$, can have a varying degree of sparseness c. However, we find that convergence properties are not affected by changing the sparseness of macroscopic state (Sup. Fig. 9). This is intuitively understood since the dimensionality of the constraint in the high-dimensional space of microscopic states is the same for all c.

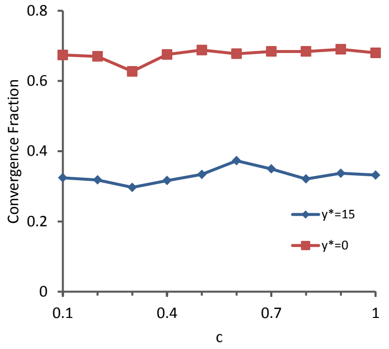

Supplementary Figure 9. Dependence of convergence fractions on the sparseness of macroscopic state vector. Convergence properties are not affected by changing the sparseness of macroscopic state, c (A). Each data point represents the fraction of network which converged within a time window of 2000 time units from a different ensemble of 500 networks. Networks in each of the ensembles have random T, J_0 and $\mathbf{x}_0$. All Ensembles have SF out-degree distribution and Binomial in-degree distribution. For all results $N = 1000$, $g_0 = 10$, $\alpha = 100$, $\mathcal{M}_0 = 2$ and $D = 10^{-3}$, $\varepsilon = 3$. SF out-degree distribution has parameters $a = 1$, $\gamma = 2.4$, and Binomial in-degree distributions has parameters $p \simeq \frac{3.5}{N}$ and N.

Dependence of Convergence on Network Motifs

Network motifs are specific local sub-graphs which are thought to be significantly over-represented in gene regulatory networks. We examined the effect of motifs on convergence by creating an ensemble of networks in which motifs are over-represented and comparing the convergence fractions of the motif-enriched networks to an appropriate null model. The single node motif of auto-regulation was discussed in the main text of the article. In this Supplementary section we shall further discuss this motif as well as motifs of higher order.

Autoregulation

the effect of auto regulation of the hubs and positive auto-regulation to random nodes was discussed in the main text. Here we examined separately the effect of adding negative ($W_{ii} < 0$) and positive ($W_{ii} > 0$) self connections. We assessed the contributions of this motif by creating an ensemble of 500 random networks of size N=1000 and adding auto regulation randomly to 10% of the nodes (Sup Fig. 10 dark green and dark blue). Such additions change the in and out degrees of some nodes in the networks and consequently affect the overall in and out degree statistics of the network. This is, in general, expected to affect convergence regardless of the auto-regulatory loops. Therefore, for each enriched network we created a null control which shares the same exact in and out degree sequence but does not include an over-representation of the auto-regulatory motif (Sup Fig. 10 light green and light blue). The control was created by randomly re-connecting out half-stubs to in half-stubs until the network is well mixed (see [2]) for details of the half-stubs method). Another control is the convergence fractions of the networks prior to any addition (Sup Fig. 10 gray). We find that both positive and negative auto regulation increases convergence, yet positive auto regulation has a considerably larger effect.

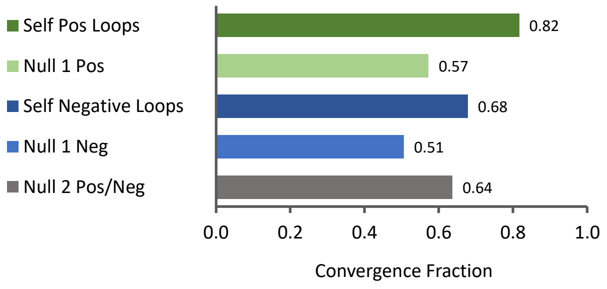

Supplementary Figure 10 Effect of adding auto-regulation loops to convergence. An ensemble of 500 networks, in which auto-regulation was added randomly to 10% of the nodes was tested for convergence. The added connections are either positive (dark green) or negative (dark blue). Results are compared to random networks with the same degree sequence (light green and light blue) and networks prior to enriching the networks with auto regulation loops (grey). Initial networks prior to the addition of auto regulation loops have SF out-degree distributions with $a = 1$, $\gamma = 2.4$ and Binomial in-degree distribution with $p = \frac{3.5}{N}$ and N. Other parameters are $N = 1000$, $g_0 = 10$, $\alpha = 100$, $\mathcal{M}_0 = 2$, $\varepsilon = 3$, $c = 0.2$, $D = 10^{-3}$ and $y* = 0$.

Feed-Forward Loops

The 3-node motif which is thought to be most significantly over-represented in regulatory networks is the feed-forward (FF) loop in which $A \Rightarrow B \Rightarrow C$ and $A \Rightarrow C$. We note that the equations governing our model are symmetric around zero and so are the connections strengths W_{ij}. Therefore there is no clear interpretation for coherent or incoherent feed-forward loops, and the signs of the connections within each motif were chosen randomly. We over-represented feed-forward loops in our networks by initially picking a random fraction of existing sequences of the form $A \Rightarrow B \Rightarrow C$, and adding to these sequences the connection $A \Rightarrow C$ which was not previously part of the network. The strengths of these added connection was drawn from the same distribution as the existing connections in the network. As in the auto-regulation motif we compare these results to a control with the same in and out degree sequence (Sup Fig. 11, blue) and to the original network prior to any addition (Sup Fig. 11, orange). Networks in the FF ensemble are enriched with 300 feed-forward loops, which increases the over-all number of FF loops by 50% on average.

We find that over-representing this motif increases convergence by 12% compared to random networks with the same degree sequences (Sup Fig. 11, FF and Null 1). However they do not contribute nor harm the convergence of the network prior to adding the loops (Sup Fig. 11, FF and Null 2).

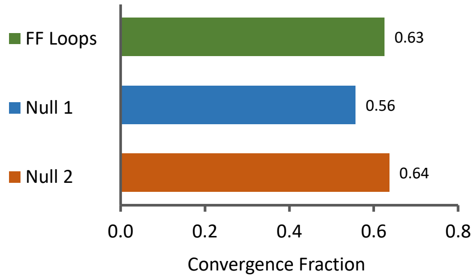

Supplementary Figure 11 Convergence of networks enriched with feed-forward loops. An ensemble of 500 networks, each enriched with 300 additional feed-forward loops, was tested for convergence (green). Results are compared to random networks with the same degree sequence (blue) and networks prior to enriching the networks with feed-forward loops (red). Initial networks prior to the addition of FF loops have SF out-degree distributions with $a = 1$, $\gamma = 2.4$ and Binomial in-degree distribution with $p = \frac{3.5}{N}$ and N. Other parameters are $N = 1000$, $g_0 = 10$, $\alpha = 100$, $\mathcal{M}_0 = 2$, $\varepsilon = 3$, $c = 0.2$, $D = 10^{-3}$ and $y* = 0$.

Bi-Fans

The four-node motivf which is thought to be most significantly over-represented in regulatory networks is the bi-fan in which two regulators jointly regulate two target genes: $A \Rightarrow C$, $A \Rightarrow D$, $B \Rightarrow C$, $B \Rightarrow D$. We find that over representing this motif does not have strong positive or negative effect compared to the network prior to adding the loops or the null model.

Stretched Exponential Fit to the Distribution of Convergence Times

Convergence times of exploratory adaptation can be well fit by a stretched exponential (main text Fig. 4). The fit is calculated by fitting $1 - log(CDF)$ of convergence times to a power law. Thus, the fit to the CDF has the stretched exponential form $1 - e^{-x/\lambda^k}$ which implies a Weibull distribution, with PDF

$$f(t) = \begin{cases} \frac{k}{\lambda}(\frac{t}{\lambda})^{k-1}e^{-t/\lambda^k} & x > 0 \\ 0 & x \leq 0 \end{cases} \tag{7}$$

For the distributions shown in the main text (Fig. 12), the fit of $1 - log(CDF)$ to a power law is excellent with $R^2 \geq 0.995$. To gain further understanding of the distribution of convergence times, we computed the empirical mean and standard deviation for increasingly larger time windows. Results show that both moments monotonically increase with the window size (even for very large time windows - Sup. Fig. 12A and 12B blue lines). In addition we calculated the stretched exponential fit for each time window. We did not use all the data points in each window, but rather a fixed number of 200 data points for all windows which were evenly distributed in the window. Thus we avoid the possibility of erroneous estimations of the quality of the fit stability which might result from an increase in the size of the data set for large time windows. Using this fit protocol we obtained excellent fits of $1 - log(CDF)$ to a power law For all time windows above t=3000 ($R^2 \geq 0.995$). We also found that the fit is stable and that after an initial transient the fit parameters fluctuate very little with increased windows sizes (Sup. Fig. 12A and 12B red lines). These findings increase our confidence in the stretched exponential fit. Moreover, although we observed an increase in mean and std with window size, they do not diverge. The stability of the fit and its large mean and std may indicate that the first two moments of the convergence times distribution are finite but can only be estimated faithfully using much larger time windows.

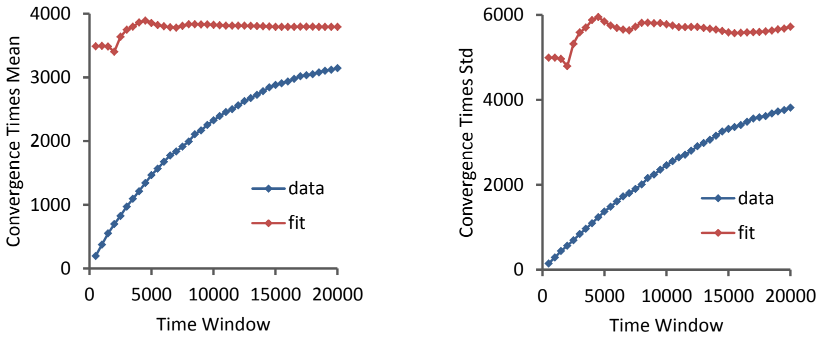

Supplementary Figure 12 Stability of stretched exponential fit to the distribution of convergence times. the empirical mean and standard deviation for increasingly larger time windows is shown (A and B blue lines). Both monotonically increase with window size for all times tested. Mean and variance estimated from parameters of the stretched exponential fit (see text for detail) are stable and after an initial transient fluctuate very little with increased windows sizes (A and B reds). Ensemble has SF out-degree distribution with $a = 1$, $\gamma = 2.4$ and Binomial in-degree distribution with $p = \frac{3.5}{N}$ and N. Other parameters are $N = 1500$, $g_0 = 10$, $\alpha = 100$, $\mathcal{M}_0 = 2$, $\varepsilon = 3$, $c = 0.2$, $D = 10^{-3}$ and $y* = 0$.

Stability of the Adapted State

As shown in the main text, a large fraction of the networks with appropriate topology converge to a stable state for which the phenotype y remains sufficiently close to the demand $y*$. From a biological point of view these final states are likely to be perturbed. Therefore it is of interest to examine the resilience of the final stable state to perturbations both in the nodes' states x_i and the interactions W_{ij}. The system at hand is high-dimensional, nonlinear and includes a stochastic feedback in the form of a random-walk in its parameters. These properties make the analytical assessment of the stability of the full model very difficult Therefore, we shall address the question of stability numerically.

Perturbations to $\mathbf{x}$

A standard linear stability analysis can be employed for network parameters W_{ij} at the values they reached following exploration. Examinations of the Jacobian matrix at such a fixed-point reveals that its eigenvalues mostly cluster around -1, with a few outliers eigenvalues (Sup Fig. 13A Inset). This is not surprising given the structure of the equation of motion $\dot{x}_i = -x_i + \sum W_{ij}\phi(x_j)$. At a fixed-point one may expect the linear term $-x_i$ to dominate typically while the other term may average out, resulting in an eigenvalue which is close to -1. The overall stability of the fixed-point can be quantified by the largest eigenvalue of the Jacobian at the fixed-point. The distribution of the largest eigenvalues, computed across and ensemble of converged networks at their respective fixed-points, are mostly located near -1 as well (Sup Fig. 13A). While such analysis provides some information as to the stability of fixed-points, it has three major disadvantages: (i) It is limited to the case where the converged state is a fixed point; (ii) It is relevant only to constant parameters W_{ij}, in contrast to the exploratory dynamics we described. Any large enough perturbation in $\mathbf{x}$ will be naturally accompanied by change in W_{ij} due to the divergence of y from the ϵ comfort-zone around $y*$. (iii) Even for constant W_ij, linear stability analysis is only valid locally around the fixed-point. For nonlinear dynamics with a large number of dimensions, the basin of attraction around a fixed point may be very small and beyond it the linear analysis does not hold. In such cases even relatively small perturbation to a system with negative eigenvalues my cause the system to lose its stability.

Given these disadvantages of linear stability analysis, we employ an additional method to assess the stability of the system to a perturbations in $\mathbf{x}$. Stability is assessed numerically by directly employing the full exploratory dynamics to perturbed variables and computing the convergence times. More specifically, we examined an ensemble of 500 networks which converged to a stable state. For each network we perturbed the final state $\mathbf{x}_{converged}$ randomly by 5%, 10%, 20%, 50%, 100% and 200%, and simulated the dynamics with the perturbed state $\mathbf{x}_0 := \mathbf{x}_{pertubed}$ and final interaction matrix $W_{converged}$ as initial condition, and the same phenotype vector $\mathbf{b}$. Convergence times for these simulation were recorded. Results are shown in Sup. Fig. 13B, alongside a control (labeled "Null") which is composed of an ensemble of 500 networks with the same parameters as the converged networks. As can be seen for perturbations of 5%, nearly all networks re-adapted within 2000 time units and a large number of these

networks re-converged very rapidly. For larger perturbation, we find a higher convergence than in the control and a lager fraction of rapid convergences. This suggests that the stability of the converged state in $\mathbf{x}$ space is non-local and that the basin of attraction covers a large area of the $\mathbf{x}$ space. Moreover, the convergence process following the perturbation involves changes of the parameters W. The rapid convergence for large number of networks suggests that the phase space of the dynamics in $\mathbf{x}$ deforms continuously with the parameters W_{ij} and that in many cases the existence of a stable attractor in not affected by small perturbations to these parameters.

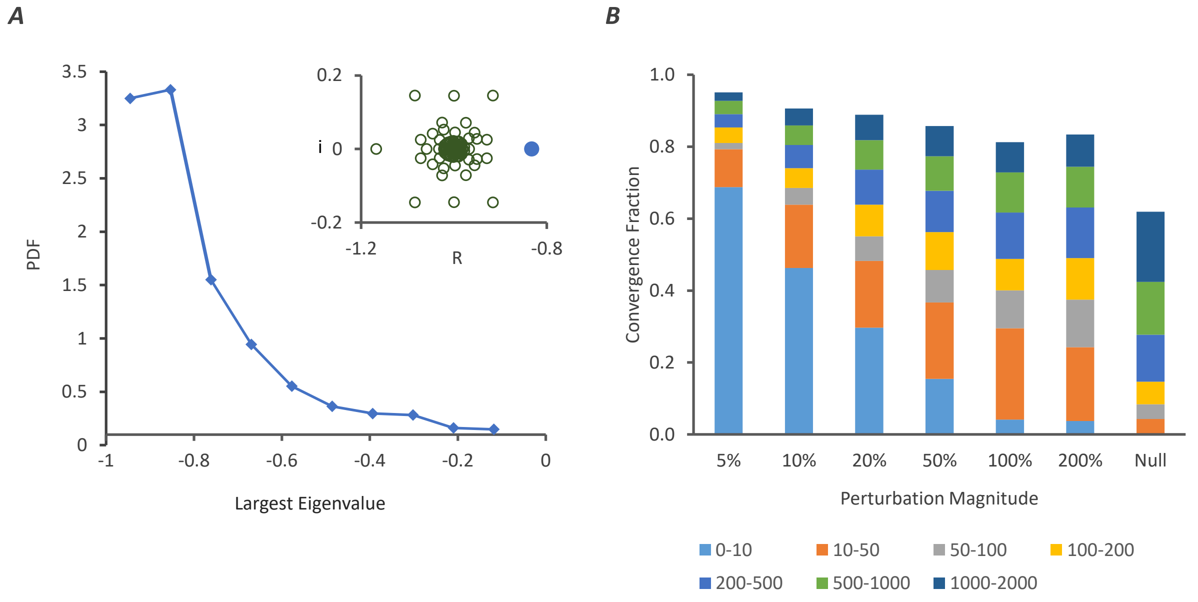

Supplementary Figure 13. Perturbation to the state vector x after convergence. (A) PDF of the maximal real-part of eigenvalues of the Jacobian matrix at the fixed-point, across an ensemble of 700 networks. Typical distribution eigenvalues in the complex plane for a single network is shown in the inset. Largest real part of eigenvalue in blue. (B) Convergence fraction of networks for which the final state $\mathbf{x}_{converged}$ was perturbed and a null control. All networks have SF out-degree distributions with $a = 1$, $\gamma = 2.4$ and Binomial in-degree distribution with $p = 3.5/N$ and N. Other parameters are $N = 1000$, $g_0 = 10$, $\alpha = 100$, $\mathcal{M}_0 = 2$, $\varepsilon = 3$, $c = 0.2$, $D = 10^{-3}$ and $y* = 0$.

Perturbations to W_{ij}

In order to asset the effect of perturbations to W_{ij} we examined 10 distinct networks $\{W^1, ..W^{10}\}$ with SF-Binom connectivity after they converged to an adapted state. For each network W^i we perturbed all non zero connections W_{ij} randomly by 1% 250 times thus obtaining 250 new networks. Simulations were then run for these 250 perturbed networks using the same phenotype vector $\mathbf{b}$ used for W^i and initial conditions $\mathbf{x}_0$ equal to the converged state $\mathbf{x}_{converged}$ of the network W^i. Re-adaptation convergence times for these perturbed networks were then recorded. This protocol was repeated for perturbations of magnitude 5%, 10%, 20% and 50%, using as a basis the same converged network W^i. In addition we constructed a null control for each converged network W^i by running 500 simulation with random choices of W_{ij} and x_i, while using the same backbone T^i which corresponds to W^i. Thus the statistics of

convergence for the perturbed networks can be compared to random networks with the same backbone T^i. All of these simulation were repeated for each of the 10 networks $\{W^1, ..W^{10}\}$. The results, averaged over the 10 networks, are shown in Sup Fig. 14. As can be seen in Sup Fig. 14A, for small perturbations of 1% nearly all networks re-adapted within 2000 time units and a large number of these networks re-converged very rapidly. For larger perturbation (5% and 10%) convergence was at lower fractions and less rapid but still more than the control (Sup. Fig. 14A.). These findings suggest a continuous picture: small perturbations re-converge rapidly in high fractions, intermediate perturbations (20%) less so, and for large perturbations (50%) convergence statistics is similar to that of random networks with the same backbone. Consistently with this picture, Sup. Fig. 14B shows that the coordinates of the new fixed-points move away from the original one in a continuous manner.

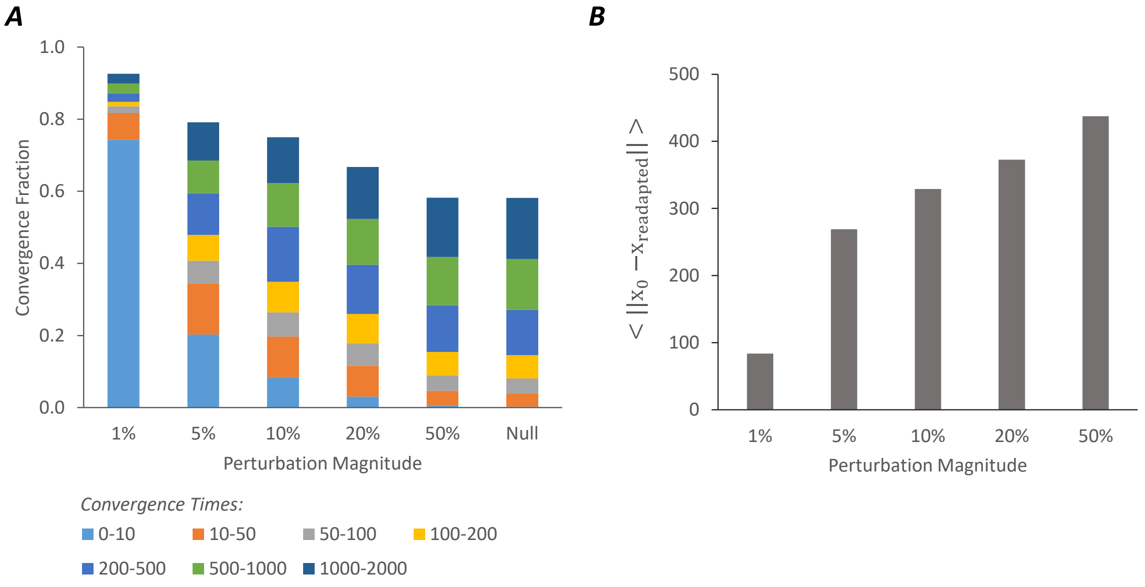

Supplementary Figure 14. Perturbation to network connection strengths after convergence. Non-zero connection strengths, W_{ij}, of 10 networks were randomly perturbed following convergence to fixed points. For each perturbation size an ensemble of 250 networks was constructed. In addition, a control ensemble was constructed consisting of 500 networks with random connection strengths W_{ij} and the same topological backbone as the original network. Results are averaged over the 10 networks.(A) Convergence fraction of perturbed networks and null control. (B) Average Euclidean distance between the initial converged state $\mathbf{x}_0$ before perturbation and the converged state after the perturbation $\mathbf{x}_{readapted}$. All networks have SF out-degree distributions with $a = 1$, $\gamma = 2.4$ and Binomial in-degree distribution with $p = 3.5/N$ and N. Other parameters are $N = 1000$, $g_0 = 10$, $\alpha = 100$, $\mathcal{M}_0 = 2$, $\varepsilon = 3$, $c = 0.2$, $D = 10^{-3}$ and $y* = 0$.

Perturbations to T

The effect of perturbations to the backbone T were assessed similarly manner to the perturbations in W_{ij} described above. We examined 10 distinct networks $\{W^1, ..W^{10}\}$ with SF-Binom connectivity after convergence; each backbone T was perturbed by adding or deleting a random fraction of connections to the network. Each such perturbation was applied 250 times and simulations were then run with the same phenotype vector $\mathbf{b}$ used for W^i. Initial conditions $\mathbf{x}_0$ for these runs were the converged state reached for

W^i prior the perturbation. For new connections $T_{ij} = 1$ that were added, the statistics of the connection strength W_{ij} was chosen randomly from the same distribution as the existing connection strengths in the network.

The results, averaged over the 10 networks, are shown in Sup Fig. 15. For both additions and deletions convergence is relatively stable for small perturbations, And for such perturbations many network quickly return to a converged state (Sup Fig. 15A and 15B).

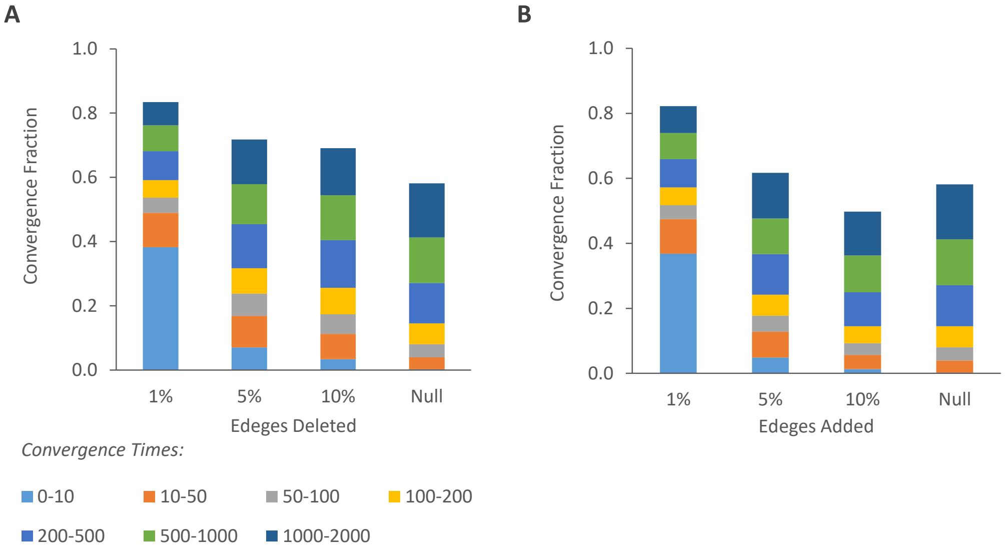

Supplementary Figure 15 Perturbation to topology of converged networks. Topology, T, of 10 converged networks was randomly perturbed by deleting and adding connections. A varying number of random connections have been removed (A) or added (B) to the converged networks. For each magnitude of perturbation an ensemble of 250 perturbed networks was constructed. Network in these ensembles were then simulated and convergence times were tracked. Results are averaged over the 10 initial networks. Initial converged networks have SF out-degree distributions with $a = 1$, $\gamma = 2.4$ and Binomial in-degree distribution with $p = 3.5/N$ and N. Other parameters are $N = 1000$, $g_0 = 10$, $\alpha = 100$, $\mathcal{M}_0 = 2$, $\varepsilon = 3$, $c = 0.2$, $D = 10^{-3}$ and $y* = 0$.

Supplementary Note 4

Dependence of Convergence of Fixed Networks on Largest Hub

We have seen that convergence of exploratory adaptation correlates with the fraction of fixed networks (constant networks and no constraint - Fig. 7 in the main text) in which the intrinsic dynamics of Eq. (1) converges to fixed points. Here we investigate further the dependence of constant networks on the network hubs. In particular, we ask whether the existence of larger hubs in a network correlates with larger probability of convergence to fixed point. In order to examine this property we randomly constructed backbones T of size N=1500 with SF out-degree and Binomial in-degree distributions. We picked 20 such backbones $\{T_1...T_{20}\}$ for which the largest hub has outgoing degrees between $K_1 \sim 100$ and $K_{20} \sim 1100$. Next we created for each backbone T_i an ensembles of 500 networks, each with random interactions strengths $\{J^{i,1}...J^{i,500}\}$, $1 \leq i \leq 20$. For each ensemble we computed the fraction of networks which converged to a fixed-point in the open-loop setting. Sup. Fig. 16 depicts this fraction as a function of the largest degree, showing a noisy but significant correlation. The large fluctuation indicate that there are other properties in addition to the largest hub that have a significant influence on convergence.

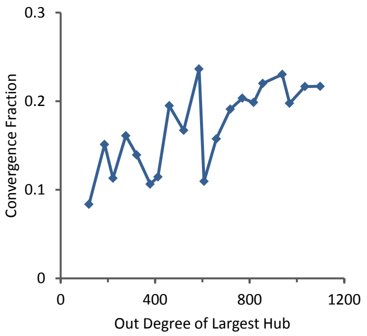

Supplementary Figure 16. Dependence of convergence fractions to fixed points in constant networks on largest hub. Twenty backbones $\{T_1...T_{20}\}$ were used to generate 20 ensembles, each composed of 500 random realizations of interaction strengths J. Each backbone T_i has a different maximal degree of the largest out-going hub, between $k_1 \sim 100$ and $k_{20} \sim 1100$. Each data point represents the fraction of networks within the ensemble which converged to a fixed-point within a time window of 2000 in the ensemble, plotted as a function of the maximal out-degree. All backbones are drawn from a SF out-degree distribution with $a = 1$, $\gamma = 2.4$ and Binomial in-degree distribution with $p \simeq \frac{3.5}{N}$ and N . $N = 1500$ and $g_0 = 10$.

Dependence of Convergence of Fixed Networks on Network Gain

Convergence fractions under exploratory adaptation dynamics are weakly dependent on the network gain g (Fig. 2 D in the main text). We examined the analogous property for constant networks by randomly constructing 5 backbones with SF out-degree and Binomial in-degree, $\{T^1...T^5\}$. For each backbone T^i we created seven ensembles of 500 networks, each with a different g, $\{(T^i, J_g^j)\}_{j=1}^{500}$, $1 \leq i \leq 5$, $g \in \{2, 3, 6, 8, 10, 12, 15\}$ (a total of 35 ensembles). For each ensemble we computed the fraction of networks for which the intrinsic dynamics converges to a fixed-point (fixed network no constraint - Sup Fig. 17 doted lines). In addition we averaged over backbones by constructing seven ensembles with $g \in \{2, 3, 6, 8, 10, 12, 15\}$ in which each network has a different T and J, $\{(T^j, J_g^j)\}_{j=1}^{500}$ (Sup. Fig. 17 dark blue). Both types of ensembles show a weak dependence on g after an initial decline for small g . In addition convergence fractions are also dependent on the specific topology T^i.

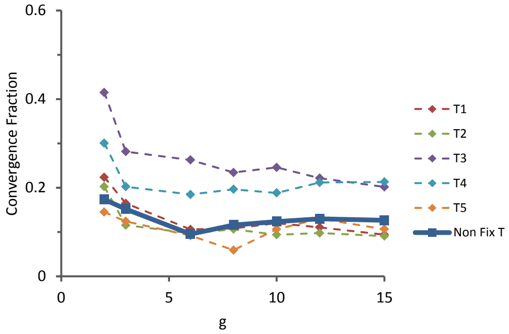

Supplementary Figure 17. Dependence of convergence fractions to fixed points in constant networks on network gain. Ensembles of 500 networks with fixed T^i, and different g, $\{(T^i, J_g^j)\}_{j=1}^{500}$, $1 \leq i \leq 5$, $g \in \{2, 3, 6, 8, 10, 12, 15\}$ (doted lines), were tested for converged to a fixed-point with a fixed network and no constraint. Mixing of the different backbones into ensembles characterized by g results in the thick blue line. Ensembles have SF out-degree distribution with $a = 1$, $\gamma = 2.4$ and Binomial in-degree distribution with $p \simeq \frac{3.5}{N}$ and N, $N = 1500$.

Supplementary References

[1] Giulia Menichetti, Luca Dall'Asta, and Ginestra Bianconi. Network controllability is determined by the density of low in-degree and out-degree nodes. *Phys. Rev. Lett.*, 113:078701, Aug 2014.

[2] Shai S Shen-Orr, Ron Milo, Shmoolik Mangan, and Uri Alon. Network motifs in the transcriptional regulation network of escherichia coli. *Nature genetics*, 31(1):64–68, 2002.
